# Time‐efficient, high‐resolution 3T whole‐brain relaxometry using Cartesian 3D MR Spin TomogrAphy in Time‐Domain (MR‐STAT) with cerebrospinal fluid suppression

**DOI:** 10.1002/mrm.30384

**Published:** 2024-11-28

**Authors:** Hongyan Liu, Edwin Versteeg, Miha Fuderer, Oscar van der Heide, Martin B. Schilder, Cornelis A. T. van den Berg, Alessandro Sbrizzi

**Affiliations:** ^1^ Computational Imaging Group, Department of Radiotheraphy University Medical Center Utrecht Utrecht The Netherlands

**Keywords:** MR‐STAT, multiparametric MRI, neuroimaging, quantitative imaging, relaxometry

## Abstract

**Purpose:**

Current three‐dimensional (3D) MR Spin TomogrAphy in Time‐Domain (MR‐STAT) protocols use transient‐state, gradient‐spoiled gradient‐echo sequences that are prone to cerebrospinal fluid (CSF) pulsation artifacts when applied to the brain. This study aims to develop a 3D MR‐STAT protocol for whole‐brain relaxometry that overcomes the challenges posed by CSF‐induced ghosting artifacts.

**Method:**

We optimized the flip‐angle train within the Cartesian 3D MR‐STAT framework to achieve two objectives: (1) minimization of the noise level in the reconstructed quantitative maps, and (2) reduction of the CSF–to–white‐matter signal ratio to suppress CSF‐associated pulsation artifacts. The optimized new sequence was tested on a gel/water phantom for accuracy evaluation of the quantitative maps, and on healthy volunteers to explore the effectiveness of the CSF artifact suppression and robustness of the new protocol.

**Results:**

An optimized sequence with high parameter‐encoding capability and low CSF signal response was proposed and validated in the gel/water phantom experiment. From in vivo experiments with 5 volunteers, the proposed CSF‐suppressed sequence produced quantitative maps with no CSF artifacts and showed overall greatly improved image quality compared with the baseline sequence. Statistical analysis indicated low intersubject and interscan variability for quantitative parameters in gray matter and white matter (1.6%–2.4% for T_1_ and 2.0%–4.6% for T_2_), demonstrating the robustness of the new sequence.

**Conclusion:**

We present a new 3D MR‐STAT sequence with CSF suppression that effectively eliminates CSF pulsation artifacts. The new sequence ensures consistently high‐quality, 1‐mm^3^ whole‐brain relaxometry within a rapid 5.5‐min scan time.

## 1 INTRODUCTION

Three‐dimensional multiparametric quantitative MRI methods have been developed to acquire multiple volumetric tissue parameters with high signal‐to‐noise ratio (SNR) efficiency from one single scan. Examples of these three‐dimensional (3D) multiparameter quantitative MRI techniques include 3D MR fingerprinting with an optimized spiral trajectory,^1^, ^2^ 3D quantification using an interleaved Look‐Locker acquisition sequence with T_2_ preparation pulse (3D‐QALAS),^3^, ^4^ quantitative transient‐state imaging,^5^, ^6^ and MR spin tomography in time domain (MR‐STAT).^7^, ^8^

MR‐STAT is a model‐based, transient‐state multiparametric quantitative MRI framework for simultaneous estimation of $T_{1}$, $T_{2}$, and proton density (PD) from a single short scan. In previous work,^9^ a 3D MR‐STAT framework was developed to achieve higher SNR compared with two‐dimensional (2D) acquisitions and isotropic resolutions for 3D volumetric quantitative maps. Specifically, the proposed 3D MR‐STAT sequence is a gradient‐spoiled, gradient‐echo (GRE) sequence using a repetitive slowly varying flip‐angle train and a regular 3D Cartesian sampling trajectory. This 3D MR‐STAT framework has been validated on healthy volunteers to acquire high‐resolution knee and bilateral lower‐leg quantitative maps. However, the previous protocol was not suitable for brain scans due to strong artifacts caused by cerebrospinal fluid (CSF) pulsation.

CSF pulsatile flow is driven by both respiratory and cardiac cycles, causing its velocity to fluctuate over time.^10^ As a result, in flow‐sensitive MR sequences like the unbalanced GRE sequence used in this study, the MR signal from CSF regions can vary in response to these velocity changes. In 3D quantitative imaging, whether using steady‐state or transient‐state SSFP (steady‐state free precession) protocols, this variation in CSF signal when sampling different phase‐encoding locations can lead to ghosting artifacts.^6^, ^9^, ^11^, ^12^ These artifacts typically originate from CSF‐rich regions, such as the cerebral ventricles, and can distort nearby brain tissues in the image domain.^13^ The artifacts become more noticeable when the CSF signal intensity is comparable to, or greater than, that of the surrounding brain structures, including periventricular white matter (WM) and deep gray matter (GM) regions like the thalami and frontal WM.

A recently developed 3D $T_{2}$ mapping protocol^14^ using a steady‐state GRE sequence incorporated a strategy to suppress the CSF signal intensity and the related ghosting artifacts. The idea is to use an optimized combination of the radiofrequency (RF) amplitudes and quadratic RF phases to achieve both good $T_{2}$ encoding for GM/WM tissues and efficient suppression for the CSF signal intensity and the related ghosting artifacts. Inspired by this method, we apply a CSF suppression strategy in our multiparametric, transient‐states MR‐STAT framework. Specifically, we optimize a time‐varying flip‐angle train for 3D MR‐STAT to simultaneously achieve (1) signal suppression for CSF and (2) high SNR T_1_ and T_2_ of GM and WM. The resulting optimized 3D MR‐STAT sequence enables whole‐brain multiparametric relaxometry in about 5.5 min at 1‐mm^3^ isotropic resolution without CSF ghosting artifacts and high quantitative image quality. Feasibility and robustness of the new protocol was validated with a water/gel phantom and 5 healthy volunteers at 3T MRI.

## 2 METHODS

### 2.1 Baseline 3D MR‐STAT sequence

MR‐STAT enables the estimation of multiparametric quantitative maps directly from transient‐state time‐domain data from one single scan. A typical 3D MR‐STAT framework uses a fast gradient‐spoiled GRE sequence with a repetitive time‐varying RF excitation flip‐angle train.^9^ A CAIPIRINHA‐pattern Cartesian undersampling trajectory^15^ can be applied to accelerate the acquisition. A waiting time, $T_{w}$, and an initial adiabatic inversion pulse are typically used at the beginning of each repetition of the flip‐angle train to achieve better T_1_ encoding. Figure S1 shows a schematic example of the 3D Cartesian MR‐STAT sequence.

**FIGURE 1:**
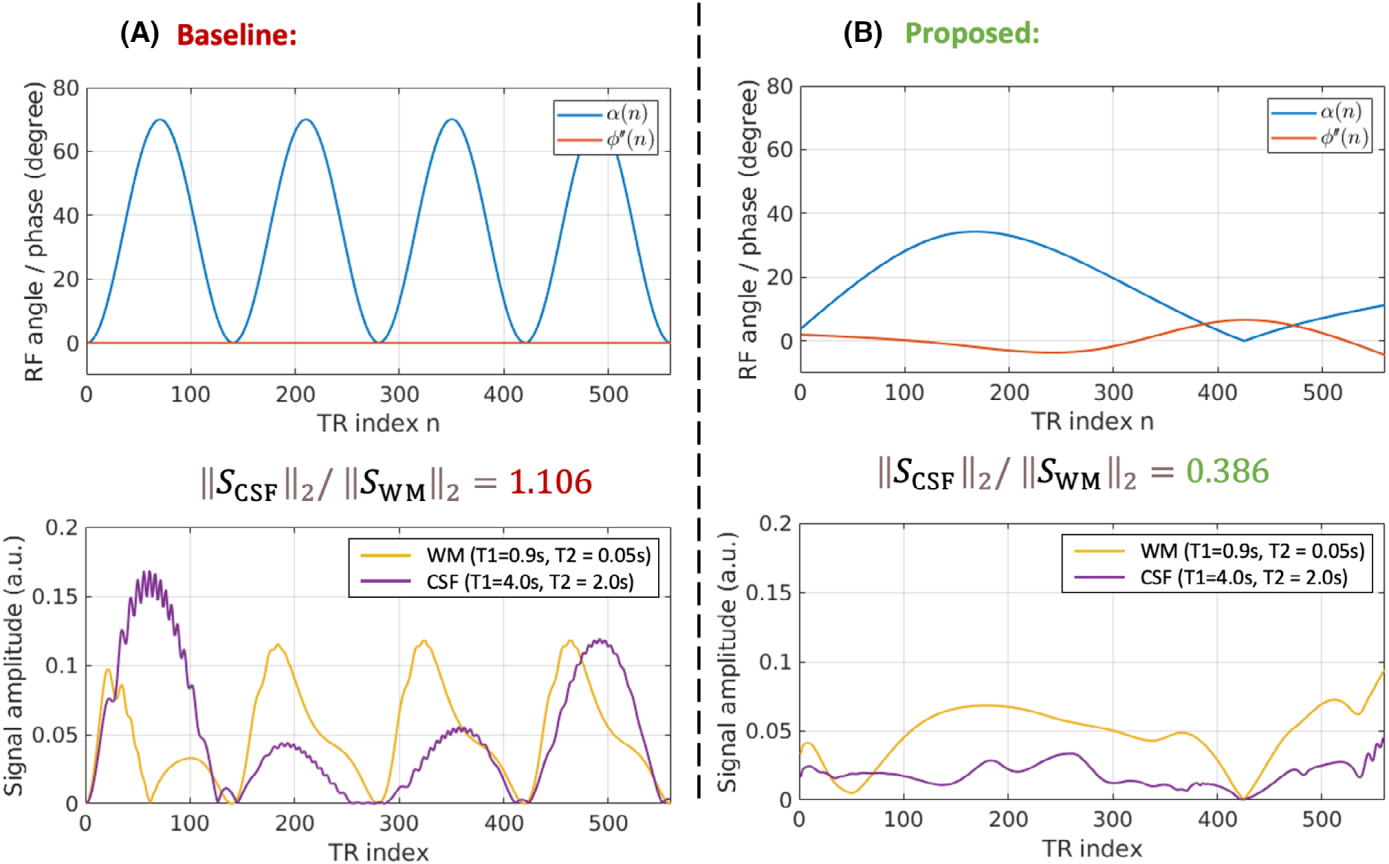
The flip‐angle train (amplitude + phase) and the simulated MR signal responses of the baseline sequence (A) and the proposed cerebrospinal fluid (CSF)–suppressed sequence (B). The corresponding simulated signals for representative CSF and white‐matter (WM) tissue values are also shown. TR, repetition time.

In previous work,^9^ a time‐varying flip‐angle train with four sine‐square lobes was used in the 3D MR‐STAT sequence. As illustrated in Figure S1, for each kz location, the modulating flip‐angle train covers the *k*_*x*_‐*k*_*y*_ plane for $N_{k}=5$ times. This acquisition uses a 3D geometry with a matrix size of $N_{x}$ (in the readout direction) $\times N_{y}$ (in the fast phase‐encoding direction) $\times N_{z}$ (in the slow phase‐encoding direction), incorporating a CAIPIRINHA pattern^15^ with an undersampling factor of 2 in both phase‐encoding directions. Consequently, this leads to the length of the flip‐angle train to be $5\times N_{y}/2$, and the number of flip‐angle train repetitions to be $N_{s}\times N_{z}/2$, where $N_{s}$ represents the slab oversampling factor in 3D acquisitions. A waiting time between repetitions of $T_{w}=1.5s$ is introduced to improve $T_{1}$ encoding. Figure 1A shows the flip‐angle train used as a baseline sequence in this work.

### 2.2 Proposed optimization of the CSF‐suppressed RF phase and amplitude modulation

Within the MR‐STAT framework, a methodology named block analysis of a k‐space‐domain Jacobian (BLAKJac)^16^ was developed to optimize the RF flip‐angle train to achieve optimal SNR in the reconstructed parameter maps. In previous work, BLAKJac has been used to optimize 2D MR‐STAT sequences, and both amplitude and quadratic RF phase modulations can be optimized simultaneously by BLAKJac to achieve better quantitative parameter encoding.^17^

In this work, we extend the use of BLAKJac to the 3D MR‐STAT framework with multiple optimization goals. Specifically, we optimize both amplitude and phase of a time‐varying flip‐angle train for a 3D MR‐STAT acquisition such that (1) the estimated noise spectrum for the reconstructed quantitative maps is low and (2) the CSF signal intensity is much lower than that of the neighboring brain tissues.

Let α(n) be the amplitude of the flip‐angle train, and $\phi^{\prime \prime }\left(n\right)$ be the second‐order phase derivative,^17^ respectively, with $n=1,2,\ldots ,N_{y}/2\times 5$. The original Cramer‐Rao bound–based BLAKJac objective function can be defined as $F_{o}\left(\alpha ,\phi^{\prime \prime }\right)$, which is a weighted sum of the noise‐level estimations for specific $\left(T_{1},T_{2}\right)$ combinations. To suppress the CSF ghosting artifacts, we add an additional term to the original objective function $F_{o}\left(\alpha ,\phi^{\prime \prime }\right)$, as follows:

(1) $$F\left(\alpha ,\phi^{\prime \prime }\right)=F_{o}\left(\alpha ,\phi^{\prime \prime }\right)+\lambda \frac{{\left\|S_{\mathrm{CSF}}\left(\alpha ,\phi^{\prime \prime }\right)\right\|}_{2}^{2}}{{\left\|S_{\mathrm{WM}}\left(\alpha ,\phi^{\prime \prime }\right)\right\|}_{2}^{2}}.$$

Here, $S_{\mathrm{CSF}}\left(\alpha ,\phi^{\prime \prime }\right)$ and $S_{\mathrm{WM}}\left(\alpha ,\phi^{\prime \prime }\right)$ represent the time‐varying signals computed by the extended phase graph model^18^ for different tissue‐parameter values, namely, $\left(T_{1},T_{2}\right)=\left(4s,2s\right)$ for $S_{\mathrm{CSF}}$ and $\left(T_{1},T_{2}\right)=\left(0.9s,0.05s\right)$ for $S_{\mathrm{WM}}$. The new objective function $F\left(\alpha ,\phi^{\prime \prime }\right)$ is optimized such that the signal norm for CSF is relatively small compared with signal norm for WM, thereby significantly reducing artifacts originating from CSF flow. The weighting parameter λ balances the CSF suppression term and the original Cramer‐Rao bound–based BLAKJac term $F_{o}\left(\alpha ,\phi^{\prime \prime }\right)$.

### 2.3 MRI experiments

#### 2.3.1 Sequence design and optimization

The following sequence parameters are used for both the baseline sequence and the proposed CSF‐suppressed sequence to be optimized by BLAKJac: matrix size $N_{x}\times N_{y}\times N_{z}=224\times 224\times 134$, resolution = 1 mm^3^, TR = 7 ms, slice oversampling factor $N_{s}=1.28$, CAIPIRINHA undersampling factor = 2 × 2. Unlike the baseline sequence where a waiting time of $T_{w}=1.5s$ is required for better $T_{1}$ encoding, a non‐stopping pattern with $T_{w}=0s$ is used for the new sequence optimization,^19^ resulting in a shorter acquisition time.

Seven different combinations of $\left(T_{1},T_{2}\right)$ pairs are used to compute the Cramer‐Rao bound–based objective term $F_{o}\left(\alpha ,\phi^{\prime \prime }\right)$, with $T_{1}$ ranging from 0.3 to 2.3 s and $T_{2}$ ranging from 0.03 to 0.2 s. The new optimized sequence with RF amplitude α(n) and phase $\phi^{\prime \prime }\left(n\right)$ is obtained by optimizing Eq. (1) using the Nelder–Mead algorithm.^20^ The values of α(n) and $\phi^{\prime \prime }\left(n\right)$ are constrained as cubic‐spline functions with five control points to reduce the search space of the optimization problem.^19^ The optimal α(n) and $\phi^{\prime \prime }\left(n\right)$ are selected from 10 repetitions of the optimization process with different randomized initial values to avoid issues of local minima after optimization. Figure S2 shows that 10 repetitions with different random initial values is sufficient for solving this highly nonconvex sequence‐optimization problem. Tuning the weighting parameter λ values can achieve different estimated noise levels and CSF‐to‐WM ratios for optimization results, as shown in Figure S3. An empirical value of λ=10 is chosen to ensure that the optimized new sequence maintains a noise level evaluated by $F_{o}\left(\alpha ,\phi^{\prime \prime }\right)$, similar to that of the baseline sequence, while minimizing the ${\left\|S_{\mathrm{CSF}}\right\|}_{2}/{\left\|S_{\mathrm{WM}}\right\|}_{2}$ signal ratio for effective CSF signal suppression.

#### 2.3.2 Phantom and in vivo experiments

The 3D MR‐STAT sequences (Figure 1) were implemented on a 3T MR scanner (Philips, The Netherlands). Both the baseline and the new optimized sequences were used for 3D scans of a phantom and in vivo brains using a 32‐channel head coil. For the baseline sequence, the total scan time was 7 min 46 s for a whole‐brain MR‐STAT acquisition. For the proposed optimized sequence, the zero waiting time $T_{w}$ led to a reduced scan time of 5 min 37 s.

Receive coil sensitivity maps were obtained using ESPIRiT^21^ from a 3D RF‐spoiled GRE sequence. The $B_{1}^{+}$ inhomogeneities were estimated using a separate fast multislice $B_{1}^{+}$ sequence^22^, ^23^ with 3.5‐mm^3^ isotropic resolution. The same field‐map protocols were used for both phantom and volunteer experiments, with parameters summarized in Table S1.

For the phantom experiments, nine gel vials with different relaxation properties (TO5, Eurospin II test system; Scotland)^24^, ^25^ and one tap‐water vial to mimic CSF were scanned using both 3D MR‐STAT sequences. For validation, gold‐standard $T_{1}$ values were acquired using inversion‐recovery spin‐echo sequences with 15 inversion times between 50 and 3800 ms, and gold‐standard $T_{2}$ values were acquired using single‐echo spin‐echo sequences with 15 echo times between 10 and 1600 ms (details in Table S2).

Five volunteers (3 male, 2 female, aged 25–35) were scanned using both sequences to assess the CSF suppression performance of the proposed new sequence. To further assess the in vivo repeatability of the proposed new sequence, 1 volunteer was repetitively scanned four times in three separate sessions. Written informed consent was obtained from all volunteers in accordance with the local institutional review board. Relaxometric statistics were computed and compared after automatically segmenting GM and WM using the FSL toolbox^26^, ^27^ based on $R_{1}\left(\frac{1}{T_{1}}\right)$ maps.

#### 2.3.3 Reconstruction

A two‐step reconstruction strategy, as described in previous work,^9^ was used for the 3D MR‐STAT reconstructions: the undersampled 3D data were first decoupled into 2D “fully sampled” MR‐STAT data using a sensitivity‐encoding (SENSE) reconstruction^28^; subsequently, slice‐by‐slice 2D MR‐STAT reconstructions were run independently along the slow phase‐encoding direction (feet–head in this case). For the first substep, an L1 wavelet regularization term was added^29^ for SENSE reconstructions of the in vivo data. For the second substep, the slice‐by‐slice 2D MR‐STAT reconstructions were performed on a desktop PC by an accelerated MR‐STAT reconstruction algorithm^30^ incorporating both a surrogate model for fast signal computations and an alternating direction method of multipliers (ADMM). A four‐layer, fully connected, complex‐valued neural network was trained to enable fast MR signal and signal derivative computations for a fixed MR‐STAT sequence, either baseline or proposed. A training data set of size 60 000 and a testing data set of size 3000 were simulated using the extended phase graph model, with the $T_{1}$ and $T_{2}$ values randomly sampled from uniform logarithmic distributions ranging from [0.1, 5] s and [0.01, 2] s, respectively. Effective $B_{1}^{+}$ values, which accounted for both imperfect slab profile and measured $B_{1}^{+}$ map, ranged from [0.4, 1.3] arbitrary units. Details of the 2D MR‐STAT reconstruction algorithm used can be found in previous work.^30^

## 3 RESULTS

### 3.1 The optimized new sequence

Figure 1 shows the flip‐angle trains of the baseline sequence^9^ (A) and the optimized sequence with CSF suppression (B). The simulated MR signals, $S_{\mathrm{CSF}}$ and $S_{\mathrm{WM}}$, for both sequences are plotted in the second row. The proposed sequence has a much lower CSF‐to‐WM signal ratio (0.386) compared with the baseline sequence (1.106), indicating an efficient suppression of the CSF. Contour plots of the signal norm for different $T_{1}$ and $T_{2}$ values are presented in Figure S4, showing suppressed signal norms when running the proposed sequence for a range of large $T_{1}$ (2.5–5 s) and $T_{2}$ (0.8–2 s) values, even though the sequence is optimized for a single ($T_{1}$, $T_{2}$) pairs of (4 s, 2 s).

### 3.2 Phantom experiment

**FIGURE 2:**
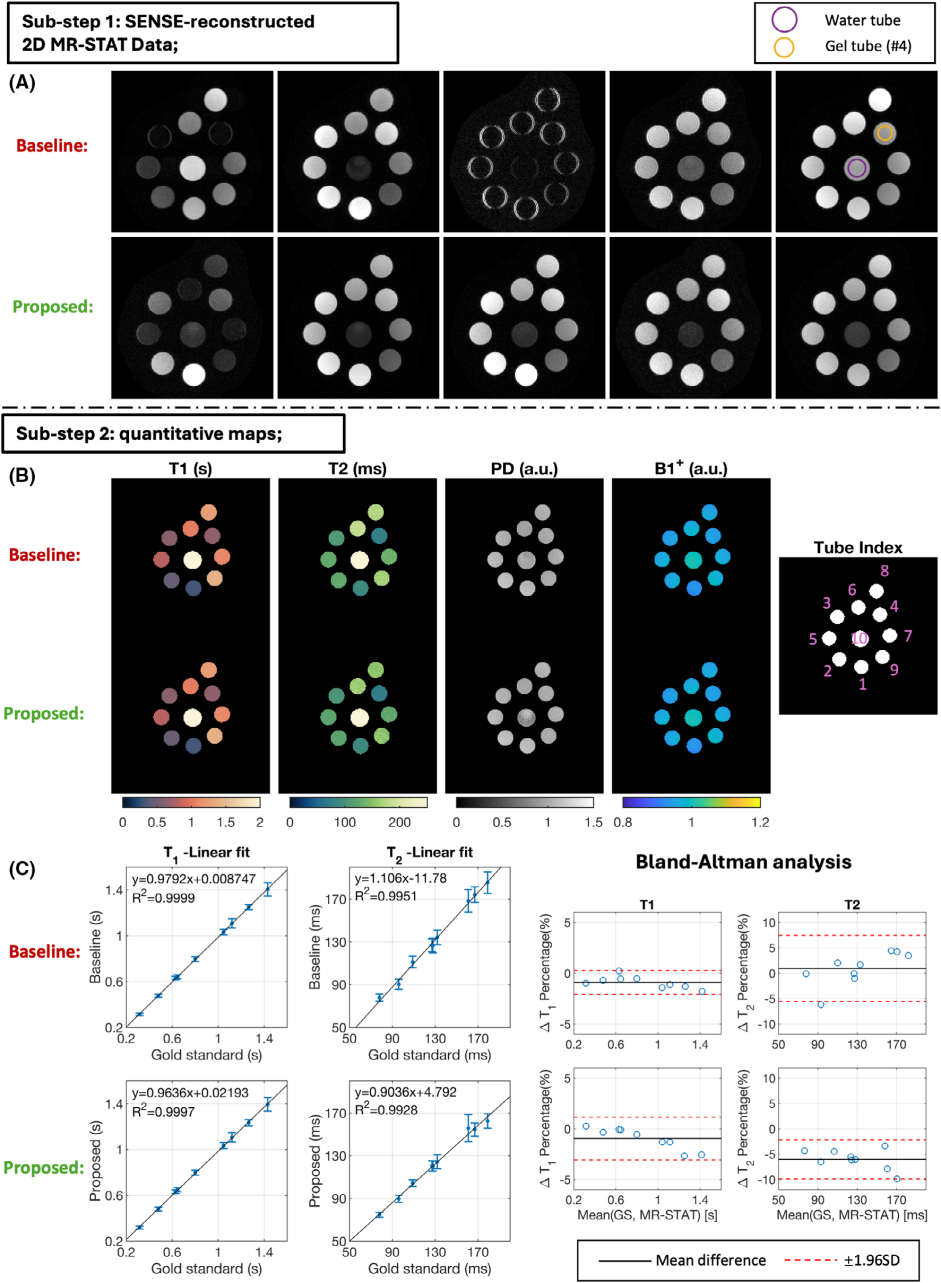
Three‐dimensional MR spin tomography in time domain (MR‐STAT) phantom results from the baseline sequence and the new optimized sequence. (A) The sensitivity‐encoding (SENSE) reconstruction results after reconstruction Substep 1. Image‐domain results of the central slice for each of the five k‐spaces are shown here for both sequences. (B) $T_{1}$, $T_{2}$, and proton density (PD) maps from both sequences after reconstruction Substep 2. Prescanned $B_{1}^{+}$ maps are also plotted. The last figure on the right shows the tube indices of the nine gel tubes (Nos. 1 to 9) and one water tube (No. 10). (C) Linear regression plot and Bland–Altman analysis plot of the quantitative results. 2D, two‐dimensional; GS, gold standard; SD, standard deviation.

Figure 2 shows the results of the water/gel‐phantom experiment. In Figure 2A, the SENSE reconstruction results of the central slice after Substep 1 are shown for both the baseline sequence and the proposed new sequence. The water tube (purple circle) is selected to mimic CSF, and the gel tube No. 4 (orange circle, ground truth $T_{1}=0.646s$ and $T_{2}=0.078s$) is selected to mimic WM tissue. A relatively low CSF‐to‐WM ratio can be observed in all five k‐spaces for the proposed new sequence, whereas relatively high CSF signals can be observed in the first, fourth, and fifth k‐spaces for the baseline sequence. The averaged CSF‐to‐WM ratio over five k‐spaces for the proposed and baseline sequence are 0.32 and 4.0, respectively. These results demonstrate the successful signal suppression for tissues/fluids with high $T_{1}$ and $T_{2}$ values.

Figure 2B,C compares the reconstructed $T_{1}$ and $T_{2}$ results from both the baseline sequence and the proposed sequence with CSF suppression. In Figure 2C, the linear regression fits for nine gel tubes show that the estimated $T_{1}$ and $T_{2}$ values from both the baseline and the proposed sequences are well aligned with the gold‐standard values. The 95% limits of agreement shown in the Bland–Altman analysis plot in Figure 2C show that the proposed sequence demonstrates higher precision in $T_{2}$ values, although slightly lower precision in $T_{1}$ values when compared with the baseline sequence. Figure S5 plots the mean and standard deviations (SDs) of $T_{1}$ and $T_{2}$ values in nine gel tubes and one water tube compared with the gold‐standard results. Table S3 summarizes the quantitative statistics of the phantom experiment, including the mean absolute error, relative SD, and sequence efficiency for both the baseline and proposed sequences. The SD values are similar for the two sequences, with a slightly higher (11.9%) $T_{1}$ relative SD observed from the proposed sequence, and a slightly higher (28.5%) $T_{2}$ relative SD observed for the baseline sequence.

### 3.3 In vivo experiments

**FIGURE 3:**
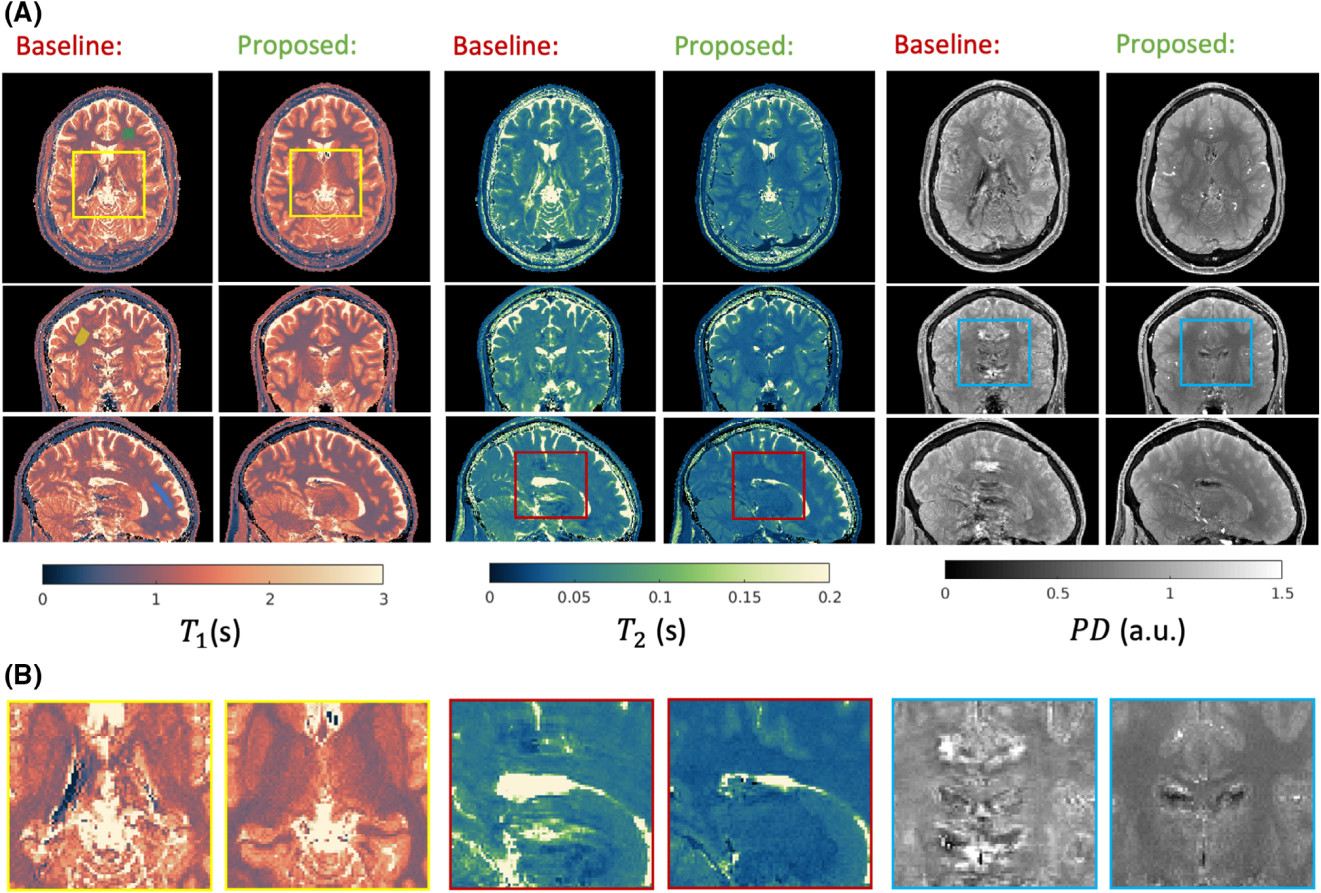
In vivo three‐dimensional MR spin tomography in time domain (MR‐STAT) results using the baseline and the proposed cerebrospinal fluid (CSF)–suppressed flip‐angle trains. Representative transverse, coronal, and sagittal slices of quantitative maps are shown for 1 healthy volunteer. Inside the zoom‐in frames, CSF ghosting artifacts can be seen for the baseline sequence reconstructions in the tissues that surround the lateral ventricle but are removed in the proposed optimized sequence with CSF suppression. Anterior–posterior: frequency‐encoding direction; left–right: fast phase‐encoding direction; feet–head: slow phase‐encoding direction. Colored shaded areas in the first column images are manually selected regions of interest with no visually obvious artifacts for signa‐to‐noise ratio evaluation (see Table S4).

Figure 3 shows in vivo 3D MR‐STAT results from both the baseline and the proposed CSF‐suppressed sequence. Representative slices of quantitative maps in all three mutually orthogonal orientations are shown for 1 volunteer. In the zoomed‐in plot in Figure 3B, CSF ghosting artifacts are observed to corrupt the baseline sequence results, particularly along the slow phase‐encoding direction (feet–head), contaminating the image quality in surrounding tissues. The proposed CSF‐suppressed sequence demonstrates greatly improved image quality for all quantitative maps. Similar figures for other volunteers are included in Figures S6–S8 and confirm these findings.

**FIGURE 4:**
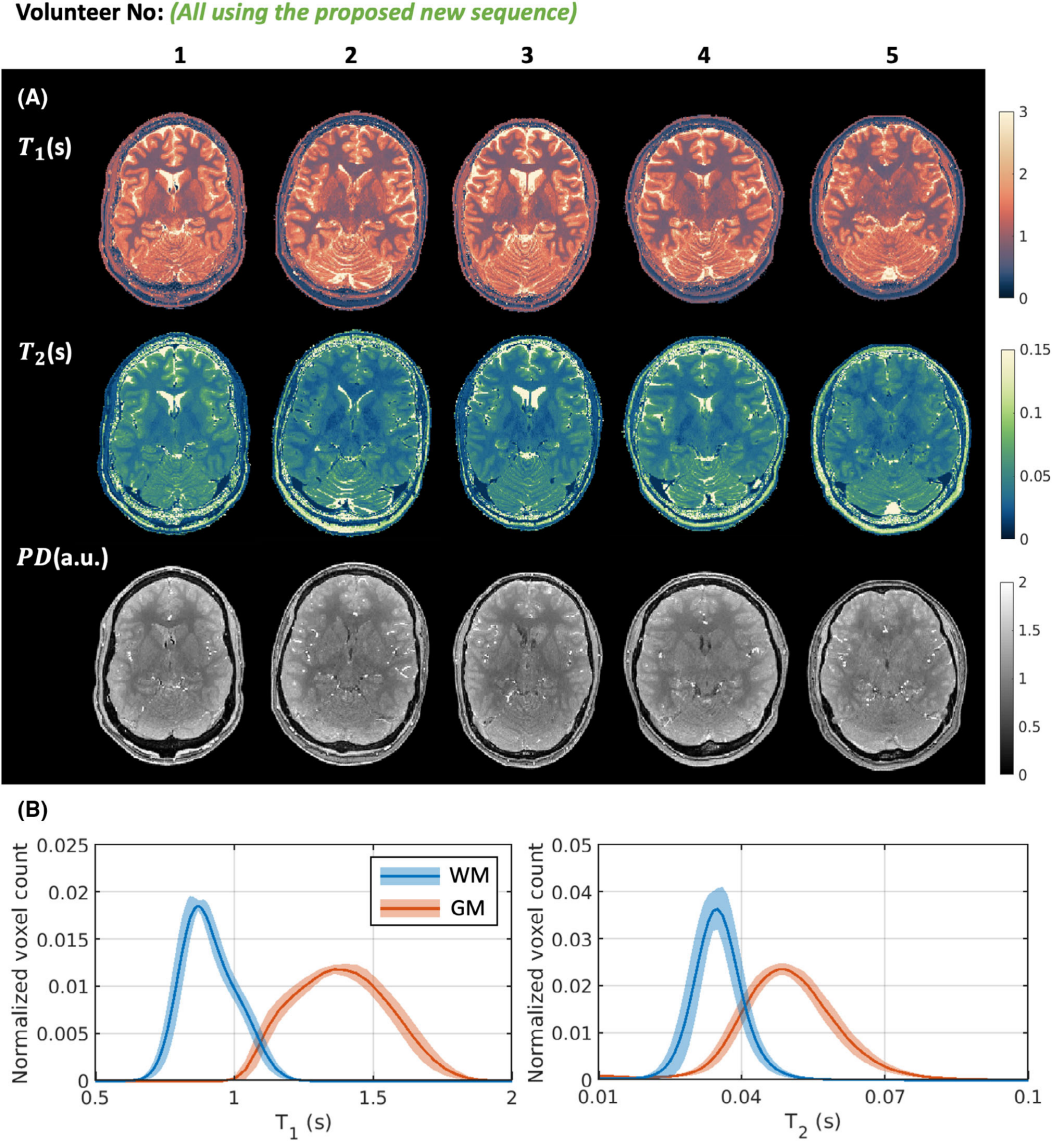
In vivo results for 5 healthy volunteers using the proposed three‐dimensional MR spin tomography in time domain (MR‐STAT) sequence with cerebrospinal fluid suppression. (A) One representative transverse slice for different volunteers. (B) Distribution of $T_{1}$ and $T_{2}$ values across different volunteers; the solid lines denote the mean values, and the light‐colored areas denote the standard deviation. GM, gray matter; PD, proton density; WM, white matter.

Figure 4 shows the in vivo results for 5 healthy volunteers using the proposed CSF‐suppressed sequence. Figure 4A shows one representative transverse slice for each of the 5 volunteers, and Figure 4B presents the whole‐brain $T_{1}$ and $T_{2}$ distributions for GM and WM of each volunteer, and the mean reconstructed $T_{1}$ and $T_{2}$ values are summarized in Table S5. The reconstructed $T_{1}$ was 912 ± 21.7 ms (2.4% intersubject variation) in WM and 1385 ± 33.6 ms (2.4% intersubject variation) in GM. The reconstructed $T_{2}$ was 35.0 ± 1.6 ms (4.6% intersubject variation) in WM and 49.7 ± 1.8 ms (3.6% intersubject variation) in GM. Overall, the observed $T_{1}$ and $T_{2}$ values fall within the range of values reported in previous literature.^19^, ^31^, ^32^, ^33^

**FIGURE 5:**
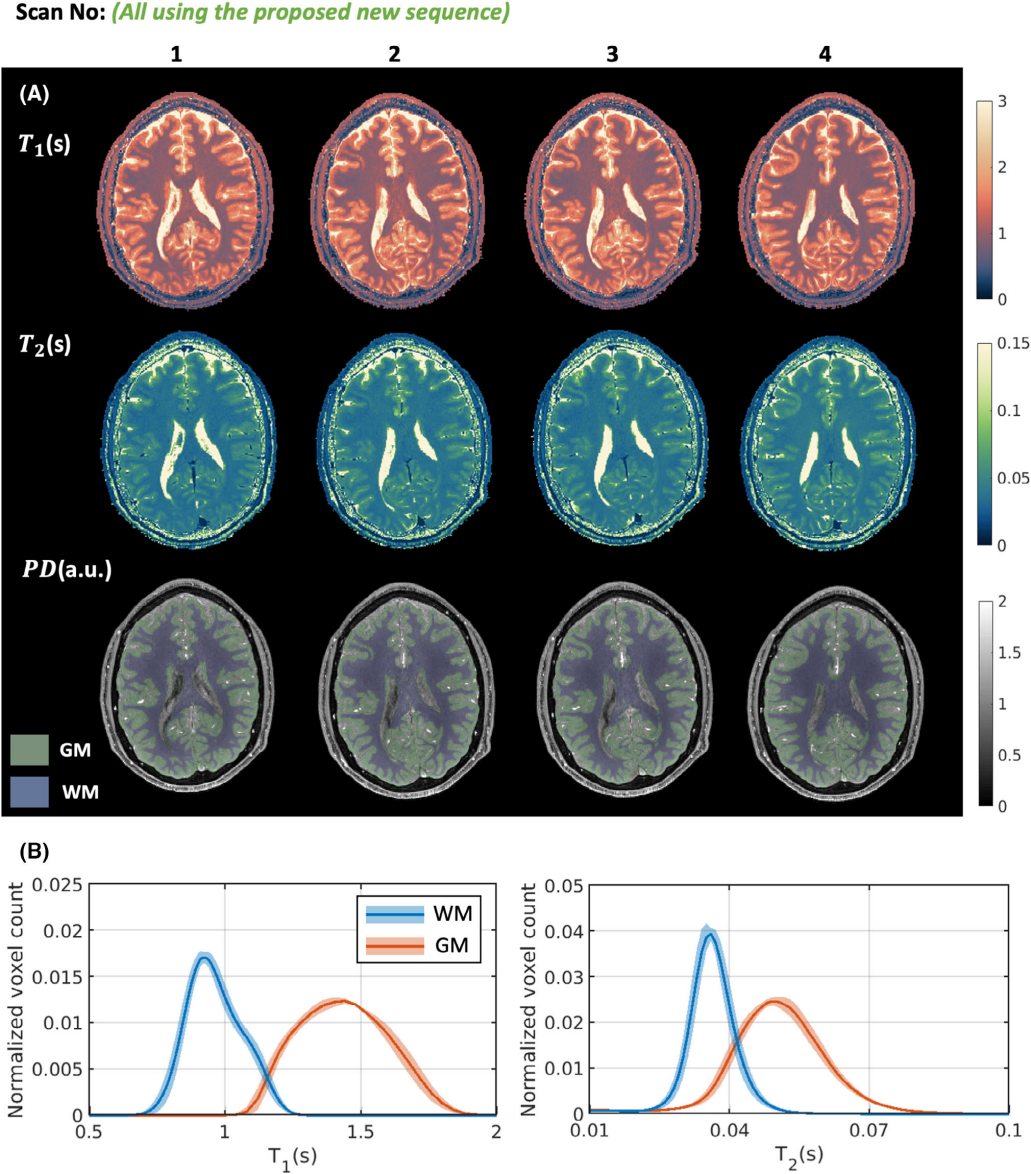
Repeatability experiment results using the proposed three‐dimensional MR spin tomography in time domain (MR‐STAT) sequence with cerebrospinal fluid suppression. One volunteer was repetitively scanned four times. The volunteer was repositioned after Scan 1 and Scan 3. (A) One representative transverse slice for different scans; gray‐matter (GM) and white‐matter (WM) segmentation results are superimposed on the proton density (PD) maps. (B) Distribution of $T_{1}$ and $T_{2}$ values across different scans; the solid lines denote the mean values, and the light‐colored areas denote the standard deviation.

Figure 5 shows repeatability results for 1 volunteer using the proposed sequence. Figure 5A shows one transverse slice for four repeating scans, and Figure 5B presents the whole‐brain $T_{1}$ and $T_{2}$ distributions for GM and WM of the four scans. Table S6 summarizes the mean reconstructed $T_{1}$ and $T_{2}$ values for WM and GM regions, along with the mean PD WM/GM ratio. The interscan variations are overall 35% lower compared with the intersubject variations: The reconstructed $T_{1}$ was 965 ± 17.7 ms (1.8% interscan variation) in WM and 1436 ± 22.3 ms (1.6% interscan variation) in GM; the reconstructed $T_{2}$ was 36.0 ± 1.0 ms (2.8% interscan variation) in WM and 50.5 ± 1.0 ms (2.0% interscan variation) in GM; and the reconstructed PD WM/GM ratio was 0.818 ± 0.0078 (1.0% interscan variation).

The in vivo results demonstrate the robustness of our proposed new sequence. Figures S11 and S12 show the whole 3D volume results for 2 different volunteers using the proposed new sequence. Reconstruction results for all volunteers are available online (https://gitlab.com/HongyanLiu/3D‐MR‐STAT‐brain).

## 4 DISCUSSION

In this work, we incorporated a CSF suppression strategy into the 3D MR‐STAT framework to mitigate CSF‐induced pulsation artifacts. This was achieved by optimizing a cyclic flip‐angle train with a mixed objective function to acquire both high SNR $T_{1}$, $T_{2}$, and PD quantitative maps and low CSF signal. Phantom experiments show that the new optimized sequence preserves high accuracy of the reconstructed quantitative maps and similar noise level compared with the baseline sequence. The higher $T_{2}$ sequence efficiency for the proposed sequence benefits from the application of quadratic RF phases, allowing for better $T_{2}$ encoding.^14^, ^34^ However, Bland–Altman analysis plot in Figure 2(C) also reveals a 6.0% underestimation of $T_{2}$ values with the proposed sequence. To further improve the precision and accuracy of the quantitative $T_{1}$ and $T_{2}$ values for the proposed sequence, future work may include more accurate signal modeling, such as considering diffusion effects and more precise modeling of RF pulses.^35^, ^36^, ^37^

The proposed 5.5‐min CSF‐suppressed sequence for whole‐brain 1‐mm^3^ isotropic relaxometry was tested on 5 healthy volunteers. Compared with the baseline sequence, the overall image quality was greatly improved due to the proposed CSF artifact‐suppression strategy. Effective CSF signal suppression of the proposed sequence is further demonstrated in Figure S9, which shows the image‐domain results after SENSE reconstruction for 1 volunteer and is consistent with the phantom results from Figure 2A. Compared with traditional contrast‐weighted images (3D T_1_‐weighted turbo spin echo and T_2_ fluid‐attenuated inversion recovery), quantitative maps from the proposed sequence show more accurate anatomical structures (Figure S10), further proving the effectiveness of the CSF artifact mitigation. Different from the phantom experiment, in which similar noise levels are observed for baseline and proposed sequences, Table S4 indicates that the proposed sequence has higher SNR values in visually artifact‐free regions for all $T_{1}$, $T_{2}$, and PD maps. This suggests that the CSF pulsation induces more perturbations in neighboring regions of the quantitative maps when running the baseline sequence, even when no obvious ghosting artifacts are visible. All these results demonstrated that the optimized cyclic sequence with locally quadratic RF phase modulation can achieve in vivo quantitative maps with both high SNR and sufficient CSF artifact suppression, leading to good image quality of the reconstructed quantitative maps.

In this work, a signal norm ratio between CSF and WM was used as the CSF‐to‐WM signal reduction term in the BLAKJac optimization problem. Although this approach reduces the ratio of signal norms between CSF and WM, it does not guarantee a consistently low CSF‐to‐WM signal ratio at every time point. Figure 1B shows that at about the 50th TR, the CSF signal amplitude is higher than the WM signal amplitude for the proposed sequence. A better signal‐reduction term may be designed in the future to further suppress such outlier conditions.

Our CSF‐suppression strategy works efficiently for quantitative neural imaging, because it relies on the relaxation time differences between CSF and other brain tissues. For in vivo brain, the GM and WM have relatively short $T_{1}$ (< 2 s) and $T_{2}$(< 0.1 s) values, whereas CSF usually has longer $T_{1}$ (typically > 3 s) and $T_{2}$ (typically > 0.2 s). This CSF‐suppression strategy based on relaxation‐time differences may be extended for other applications, such as blood signal suppression for cardiovascular imaging,^38^ and these possibilities will be explored and investigated in the future.

In addition to the proposed CSF signal intensity suppression strategy, there are other methods being developed to suppress pulsatile flow artifacts. For example, flow‐compensation techniques by adjusting imaging gradients can be applied to reduce the signal's phase contribution caused by flow.^39^, ^40^, ^41^ Another recently developed technique designs a randomized order of phase encoding to eliminate the repeating artifacts induced by semi‐periodic physiological signal fluctuations.^12^ Future work may also include the comparison or combined application of these methods with the proposed CSF‐suppression method.

In this work, the proposed 3D MR‐STAT sequence for whole‐brain relaxometry has been validated on healthy volunteers. Numerous studies have demonstrated that alterations in quantitative tissue parameters are linked to neurological diseases, including multiple sclerosis, epilepsy, and Parkinson's disease.^42^, ^43^, ^44^, ^45^, ^46^ We are confident that our protocol holds significant promise for clinical research, and advancing the application of our technique will be a primary focus of our future work.

## 5 CONCLUSION

We demonstrated a new Cartesian 3D MR‐STAT sequence with inherent CSF suppression that mitigates the CSF pulsation artifacts and consistently obtained high‐quality, 1‐mm^3^ whole‐brain relaxometry within a 5.5‐min scan time.

## Supporting information


**Data S1.** Supporting information.
**Figure S1.** Schematic example of a three‐dimensional (3D) Cartesian MR spin tomography in time domain (MR‐STAT) sequence with a CAIPIRINHA sampling factor of 2 x 2 in *k*_*y*_‐*k*_*z*_ phase‐encoding plane.
**Figure S2.** The objective function (α,″) for sequence optimization problem (1) using different numbers of repetitions with different random initial values.
**Figure S3.** Estimated noise level (α,″) and the cerebrospinal fluid (CSF) signal ratio ‖*S*_CSF_‖_2_/‖*S*_WM_‖_2_ for sequence‐optimization results using different *λ* values.
**Figure S4.** Contour plot of the signal norm for different *T*_1_ and *T*_2_ values.
**Figure S5.** Mean and standard deviations of *T*_1_ and *T*_2_ values in nine gel tubes and one water tube compared with the gold‐standard results in the phantom experiment.
**Figure S6.** In vivo three‐dimensional (3D) MR spin tomography in time‐domain (MR‐STAT) results using the baseline and the new optimized flip‐angle trains.
**Figure S7.** In vivo three‐dimensional (3D) MR spin tomography in time‐domain (MR‐STAT) results using the baseline and the new optimized flip‐angle trains.
**Figure S8.** In vivo three‐dimensional (3D) MR spin tomography in time‐domain (MR‐STAT) results using the baseline and the new optimized flip‐angle trains.
**Figure S9.** Sensitivity‐encoding (SENSE) reconstruction results after reconstruction Substep 1 for Volunteer 1.
**Figure S10.** In vivo three‐dimensional (3D) MR spin tomography in time‐domain (MR‐STAT) results using the baseline and the proposed cerebrospinal fluid (CSF)–suppressed flip‐angle trains.

**Figure S11.** Animation of whole three‐dimensional (3D) volume quantitative maps for Volunteer 1 using the proposed new sequence. (See separately uploaded mp4 file.)

**Figure S12.** Animation of whole three‐dimensional (3D) volume quantitative maps for Volunteer 6 using the proposed new sequence. Conventional contrast‐weighted images (3D T_1_‐weighted turbo spin echo [T1w‐TSE] and T_2_‐weighted fluid‐attenuated inversion recovery [T2‐FLAIR]) are shown in the second row for reference. (See separately uploaded mp4 file.)
**Table S1.** Non–MR spin tomography in time domain (MR‐STAT) sequences used for estimating coil sensitivity maps and B_1_^+^ maps.
**Table S2.** Gold‐standard T_1_ and T_2_ mapping protocols used for phantom experiments.
**Table S3.** Quantitative statistics of the phantom experiment.
**Table S4.** Computed signal‐to‐noise ratio (SNR) in manually selected in vivo region‐of‐interest (ROI) regions in Figure 3 for both the baseline and the proposed sequences.
**Table S5.** Reconstructed *T*_1_ and *T*_2_ values from 5 different volunteers.
**Table S6.** Reconstructed *T*_1_, *T*_2_, and proton‐density (PD) values from the repeatability experiment.

## Data Availability

The 3D quantitative reconstruction results for all 5 volunteers are available on the open‐access GitLab repository (https://gitlab.com/HongyanLiu/3D‐MR‐STAT‐brain).
