# Supplementary material for: Time‐efficient, high‐resolution 3T whole‐brain relaxometry using Cartesian 3D MR Spin TomogrAphy in Time‐Domain (MR‐STAT) with cerebrospinal fluid suppression
Source: Magn Reson Med. 2024 Nov 28;93(5):2008–19. doi: 10.1002/mrm.30384 (PMC11893030; doi:10.1002/mrm.30384)
Supplement: Supplementary file 1 — Data S1. Supporting information. Figure S1. Schematic example of a three‐dimensional (3D) Cartesian MR spin tomography in time domain (MR‐STAT) sequence with a CAIPIRINHA sampling factor of 2 x 2 in k y ‐k z phase‐encoding plane. Figure S2. The objective function (α,″) for sequence optimization problem (1) using different numbers of repetitions with different random initial values. Figure S3. Estimated noise level (α,″) and the cerebrospinal fluid (CSF) signal ratio ‖S CSF‖2/‖S WM‖2 for sequence‐optimization results using different λ values. Figure S4. Contour plot of the signal norm for different T 1 and T 2 values. Figure S5. Mean and standard deviations of T 1 and T 2 values in nine gel tubes and one water tube compared with the gold‐standard results in the phantom experiment. Figure S6. In vivo three‐dimensional (3D) MR spin tomography in time‐domain (MR‐STAT) results using the baseline and the new optimized flip‐angle trains. Figure S7. In vivo three‐dimensional (3D) MR spin tomography in time‐domain (MR‐STAT) results using the baseline and the new optimized flip‐angle trains. Figure S8. In vivo three‐dimensional (3D) MR spin tomography in time‐domain (MR‐STAT) results using the baseline and the new optimized flip‐angle trains. Figure S9. Sensitivity‐encoding (SENSE) reconstruction results after reconstruction Substep 1 for Volunteer 1. Figure S10. In vivo three‐dimensional (3D) MR spin tomography in time‐domain (MR‐STAT) results using the baseline and the proposed cerebrospinal fluid (CSF)–suppressed flip‐angle trains. [file MRM-93-2008-s003.pdf]

Time-efficient, high-resolution 3T whole-brain relaxometry using Cartesian 3D MR-STAT with CSF suppression

Supplementary material

Table S1. Non-MR-STAT sequences used for estimating coil sensitivity maps and B_1^+ maps. The same sequences were used for both phantom and in vivo experiments, with a total scan time of approximately 44 seconds. Smooth, high-resolution sensitivity maps (matching the 3D MR-STAT resolution) were acquired using the ESPIRiT method with zero-filled k-space data. High-resolution B_1^+ maps were obtained by solving a model-based problem with an L2 norm of the image's Laplacian operator.

<table>
<tr><td rowspan="2">Receive coil sensitivity maps</td><td>Sequence Type</td><td>TR (ms)</td><td>TE (ms)</td><td>Flip angle (degree)</td><td>Matrix size</td><td>Voxel Size (mm^3)</td><td>Scan time (sec)</td></tr>
<tr><td>3D RF-spoiled gradient-echo sequence</td><td>5.7</td><td>2.7</td><td>20</td><td>64 x 64 x 60</td><td>3.5 x 3.5 x 3.5</td><td>27.8</td></tr>
<tr><td rowspan="2">B_1^+ maps</td><td>Sequence Type</td><td>TR (ms)</td><td>TE1/TE2 (ms)</td><td>Flip angle (degree)</td><td>Matrix size</td><td>Voxel Size (mm^3)</td><td>Scan time (sec)</td></tr>
<tr><td>Multi-slice 2D $B1^+$ DREAM sequence [1-3]</td><td>4.6</td><td>1.8/2.4</td><td>40</td><td>64 x 64 x 40</td><td>3.5 x 3.5 x 3.5</td><td>16</td></tr>
</table>

Reference

[1] M. Uecker, P. Lai, M. J. Murphy, P. Virtue, M. Elad, J. M. Pauly, S. S. Vasanawala, and M. Lustig, "ESPIRiT—an eigenvalue approach to autocalibrating parallel MRI: Where SENSE meets GRAPPA," *Magn Reson Med*, vol. 71, no. 3, pp. 990–1001, Mar. 2014.

[2] K. Nehrke, M. J. Versluis, A. Webb, and P. Börnert, "Volumetric B1+ mapping of the brain at 7T using DREAM," *Magn Reson Med*, vol. 71, no. 1, pp. 246–256, 2014.

[3] Nehrke, K., & Börnert, P. (2012). DREAM—a novel approach for robust, ultrafast, multislice B1 mapping. *Magnetic resonance in medicine*, *68*(5), 1517-1526.

Table S2. Gold standard T1 and T2 mapping protocols used for phantom experiments.
We choose inversion-recovery spin-echo sequences with different TIs for T1 mapping protocol, and single-echo spin-echo sequences with different TEs for T2 mapping protocol. Since both sequence types require relatively long scan time, usually a few hours in total for acquiring a single 2D slice, we modify the protocol to run one-dimensional sequences to reduce total scan time. These 1D measurement sequences sacrifice the spatial resolution (assuming homogeneous gel tubes), require only one phase-encoding line for each repetition, but reduce the total scan time to less than 5 minutes.

**T1 mapping protocol**	**Sequence Type**	**TR (ms)**	**TI (ms)**	**Matrix size**	**Voxel Size (mm^3)**	**Scan time (sec)**
	A series of inversion-recovery spin-echo sequences with different TIs	10000	[50; 70; 95; 130; 170; 235; 320; 440; 600; 810; 1100; 1500; 2050; 2800; 3800]	224 x 1 x 1	1 x 224 x 5	150
**T2 mapping protocol**	**Sequence Type**	**TR (ms)**	**TE (ms)**	**Matrix size**	**Voxel Size (mm^3)**	**Scan time (sec)**
	A series of single-echo spin-echo sequences with different TEs	5000	[10; 14; 20; 30; 43; 60; 88; 127; 180; 260; 380; 540; 780; 1140; 1600]	224 x 1 x 1	1 x 224 x 5	75

Table S3. Quantitative statistics of the phantom experiment. The standard deviation (SD) values are similar for the two sequences, with a slightly higher (11.9%) T_1 relative SD observed from the proposed sequence, and a slightly higher (28.5%) T_2 relative SD observed for the baseline sequence. When considering the sequence efficiency, defined as the $T_{1,2}$ SNR divided by the square root of the scan time per slice , we find a T_1 sequence efficiency of 18.8 for the baseline sequence and 19.8 (4.9% higher) for the proposed sequence. The T_2 sequence efficiency is 9.7 for the baseline sequence and 14.7 (50.9% higher) for the proposed new sequence.

	**MAE (Mean Absolute Error)**		**Relative SD**		**Sequence efficiency**	
	Baseline	Proposed	Baseline	Proposed	Baseline	Proposed
T1	9.4ms	11.7ms	0.0280	0.0313	18.8	19.8
T2	3.6ms	7.9ms	0.0531	0.0413	9.7	14.7

Table S4. Computed SNR in manually selected in-vivo ROI regions in **Figure 3** for both the baseline and the proposed sequences.

**SNR**	**T_1(ms)**		**T_2(ms)**		**PD (a.u.)**	
	Baseline	Proposed	Baseline	Proposed	Baseline	Proposed
ROI 1	10.4	22.8	8.3	13.5	16.9	20.2
ROI 2	16.1	23.6	10.6	14.1	14.7	20.2
ROI 3	14.3	21.1	10.2	15.0	16.6	25.1
Mean over 3 ROIs	13.6	22.5	9.7	14.2	16.1	21.8

Table S5. Reconstructed T_1 and T_2 values from five different volunteers. See also **Figure 4** for example image slices and histogram plots. Literature values are from previous reference [1-4].

	T_1(ms)		T_2(ms)	
	WM	GM	WM	GM
Volunteer1	927	1416	36.7	52.1
Volunteer2	902	1392	32.5	47.7
Volunteer3	935	1416	34.3	48.9
Volunteer4	880	1347	36.0	50.9
Volunteer5	914	1352	35.4	48.9
$\mu \pm \sigma$	912 ± 21.7	1385 ± 33.6	35.0 ± 1.6	49.7 ± 1.8
Literature	728 - 1110	1165 - 1820	29 - 56	43 - 99

[Reference]

[1] J. Assländer, R. Lattanzi, D. K. Sodickson, and M. A. Cloos, "Optimized quantification of spin relaxation times in the hybrid state," *Magn Reson Med*, vol. 82, no. 4, pp. 1385–1397, Oct. 2019.

[2] J. Z. Bojorquez, S. Bricq, C. Acquitter, F. Brunotte, P. M. Walker, and A. Lalande, "What are normal relaxation times of tissues at 3 T?," Magnetic Resonance Imaging, vol. 35. Elsevier Inc., pp. 69–80, 01-Jan-2017.

[3] G. J. Stanisz, E. E. Odrobina, J. Pun, M. Escaravage, S. J. Graham, M. J. Bronskill, and R. M. Henkelman, "T1, T2 relaxation and magnetization transfer in tissue at 3T," Magn Reson Med, vol. 54, no. 3, pp. 507–512, 2005.

[4] J. Y. Choi, S. Hu, T. Y. Su, H. Murakami, Y. Tang, I. Blümcke, I. Najm, K. Sakaie, S. Jones, M. Griswold, Z. I. Wang, and D. Ma, "Normative quantitative relaxation atlases for characterization of cortical regions using magnetic resonance fingerprinting," Cerebral Cortex, vol. 33, no. 7, pp. 3562–3574, Apr. 2023.

Table S6. Reconstructed T_1, T_2, and PD values from the repeatability experiment. See also **Figure 5** for example image slices and histogram plots. Mean T_1 and T_2 values for WM and GM regions, along with the mean PD WM/GM ratio, are reported for the four different scans.

		T_1(ms)		T_2(ms)		PD
		WM	GM	WM	GM	WM/GM Ratio
Session1	Scan1	975	1445	35.0	49.8	0.823
Session2	Scan2	974	1446	35.9	50.1	0.822
	Scan3	971	1451	35.8	50.1	0.820
Session3	Scan4	938	1403	37.4	52.0	0.806
$\mu \pm \sigma$		965 ± 17.7	1436 ± 22.3	36.0 ± 1.0	50.5 ± 1.0	0.818 ± 0.0078
CoV		1.83%	1.55%	2.78%	2.00%	0.95%

Figure S1. Schematic example of a 3D Cartesian MR-STAT sequence with a CAIPIRINHA sampling factor of 2 x 2 in ky-kz phase encoding plane. The yellow trajectory shows the sampling order. An initial adiabatic inversion pulse is used at the beginning of each repetition of the flip-angle train, and a waiting time N_w is used between repetitions.

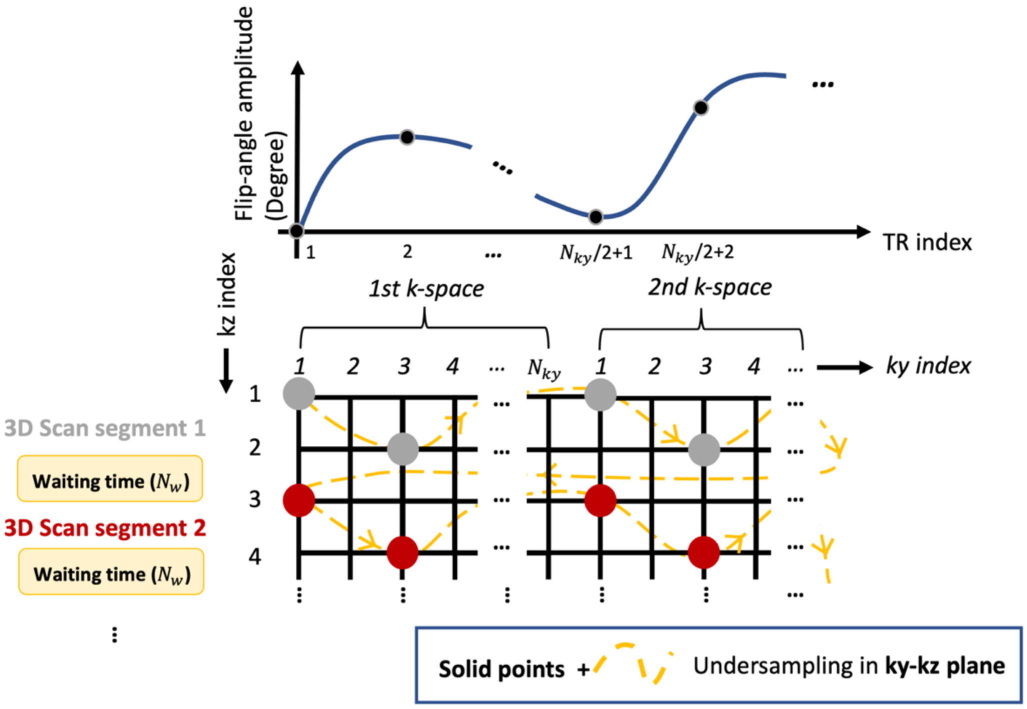

Figure S2. The objective function $F(\alpha, \phi'')$ for sequence optimization problem (1) using different numbers of repetitions with different random initial values.

To demonstrate 10 repetitions are sufficient, we repeated the sequence optimization process N times ($N = 1, 2, 5, 10, 20, 50, 100$) using different initialization states each time, and plotted the minimum $F(\alpha, \phi'')$ observed within N repetitions. While we cannot guarantee that the result obtained after 10 repetitions is the global optimal solution, it can be observed that after about 5 repetitions, the reduction of the objective function $F(\alpha, \phi'')$ becomes relatively slow and almost negligible. For robustness, we used 10 repetitions as an empirical value, and demonstrated that the optimized sequence effectively preserves good SNR while reducing the CSF signal.

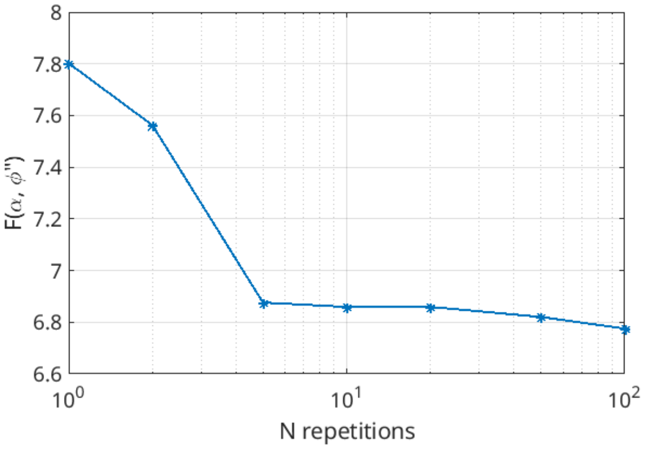

Figure S3. Estimated noise level $F_o(\alpha, \phi'')$ and the CSF signal ratio $\|S_{CSF}\|_2 / \|S_{WM}\|_2$ for sequence optimization results using different λ values. The dashed lines represent the noise level and CSF signal ratio for the Baseline sequence. It can be observed that with $\lambda = 10$, the estimated noise level is lower than Baseline level, and the CSF signal ratio is the lowest for $\lambda \leq 10$. This suggests that an empirical value of $\lambda = 10$ maintains a good balance between SNR and CSF suppression.

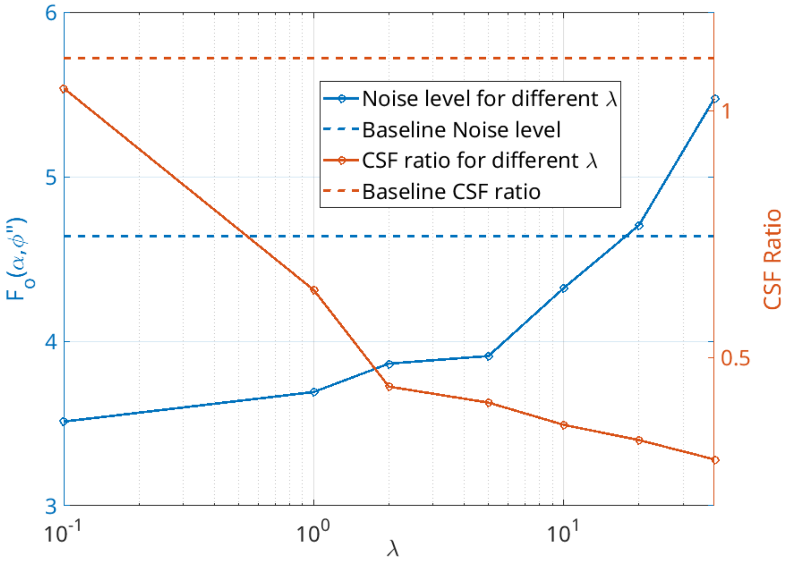

Figure S4. Contour plot of the signal norm for different T1 and T2 values. The signal norms for relatively large T1 (2.5s – 5s) and T2 (0.8s – 2s) values (dashed blue box at the top right corner) are suppressed for the proposed sequence but remain relatively large values for the baseline sequence. The back regions are cut-off regions of unrealistic combinations with T1< T2. Note that both axes are plotted in a logarithmic scale.

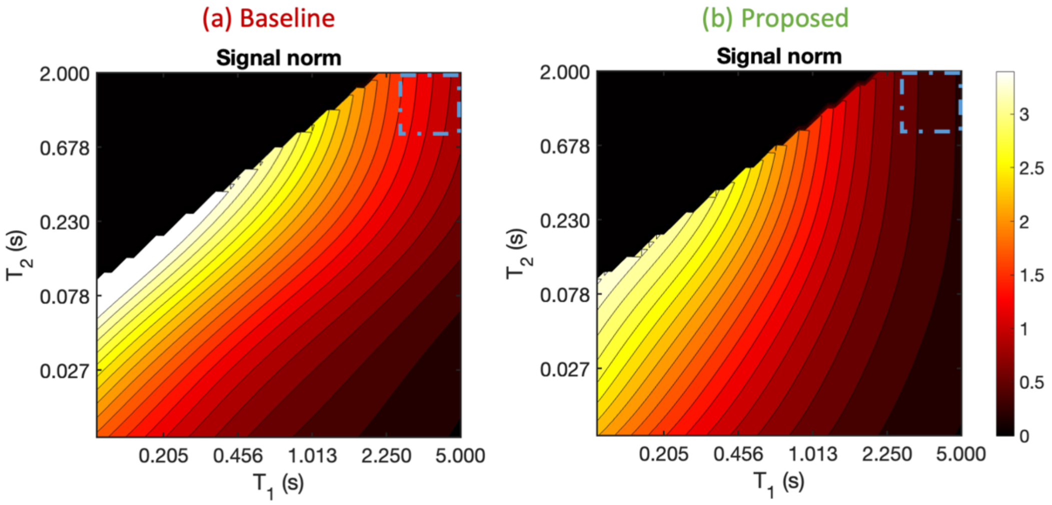

Figure S5. Mean and standard deviations of T_1 and T_2 values in nine gel tubes and one water tube compared to the gold standard results in the phantom experiment.

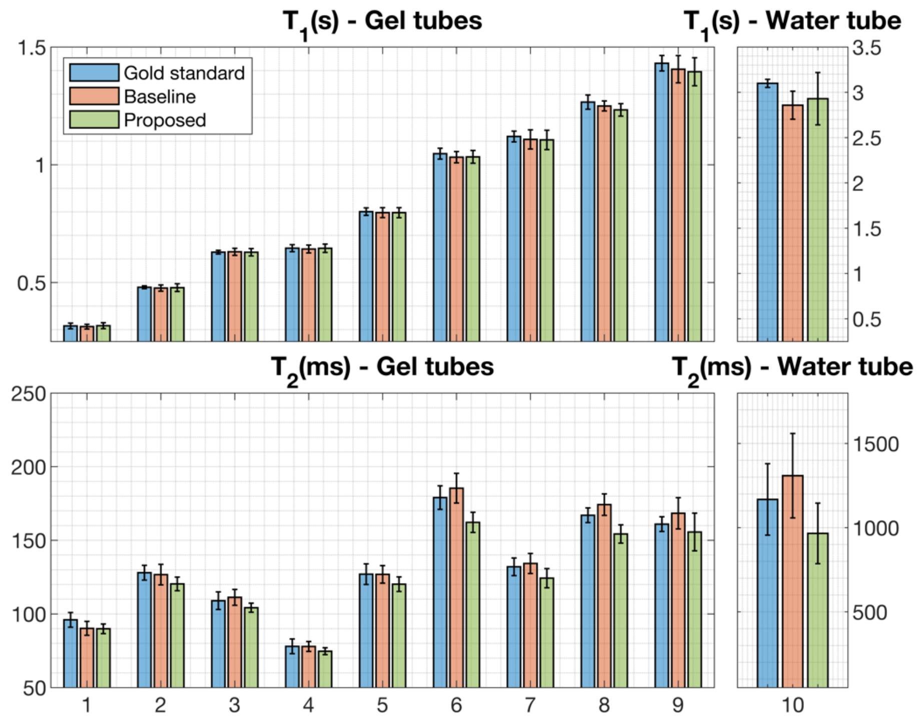

Figure S6. In-vivo 3D MR-STAT results using the Base-line and the new optimized flip-angle trains. Similar as Figure 3 but for volunteer no. 2.

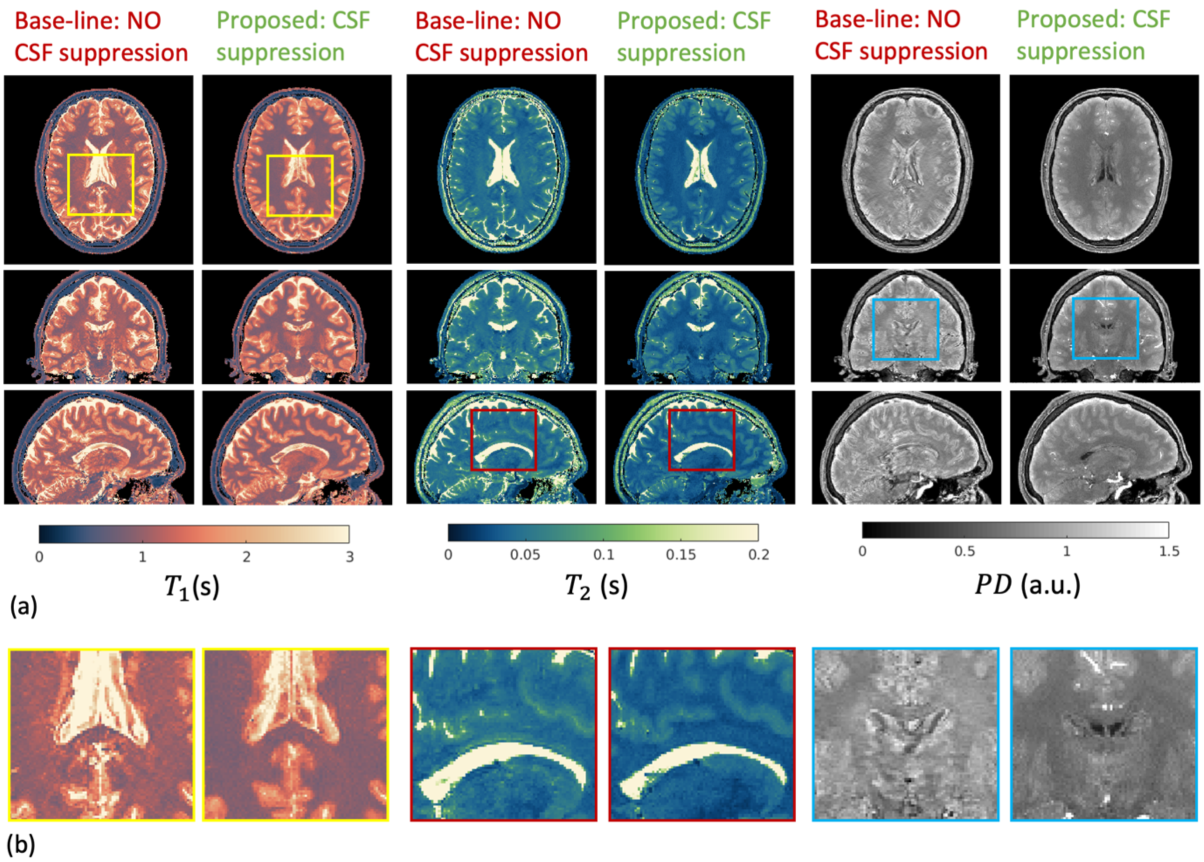

Figure S7. In-vivo 3D MR-STAT results using the Base-line and the new optimized flip-angle trains. Similar as Figure 3 but for volunteer no. 3.

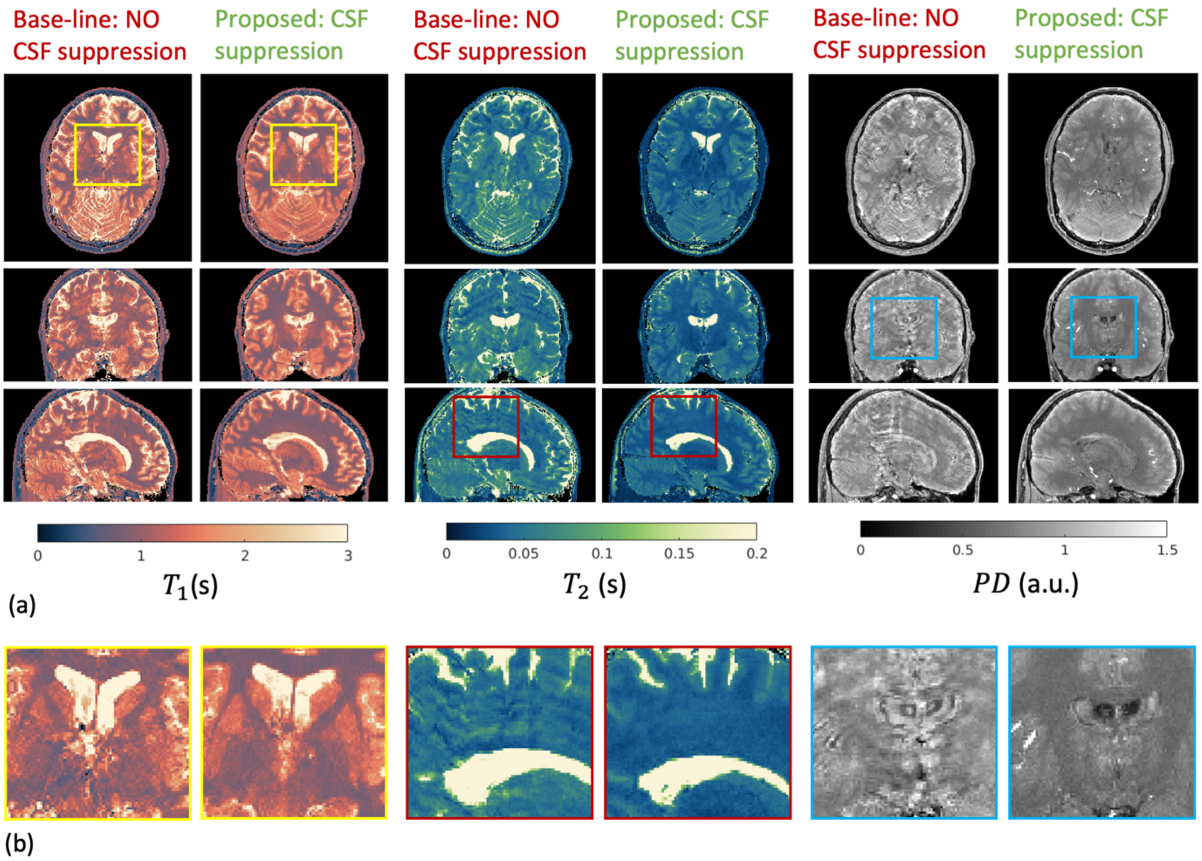

Figure S8. In-vivo 3D MR-STAT results using the Base-line and the new optimized flip-angle trains. Similar as Figure 3 but for volunteer no. 4.

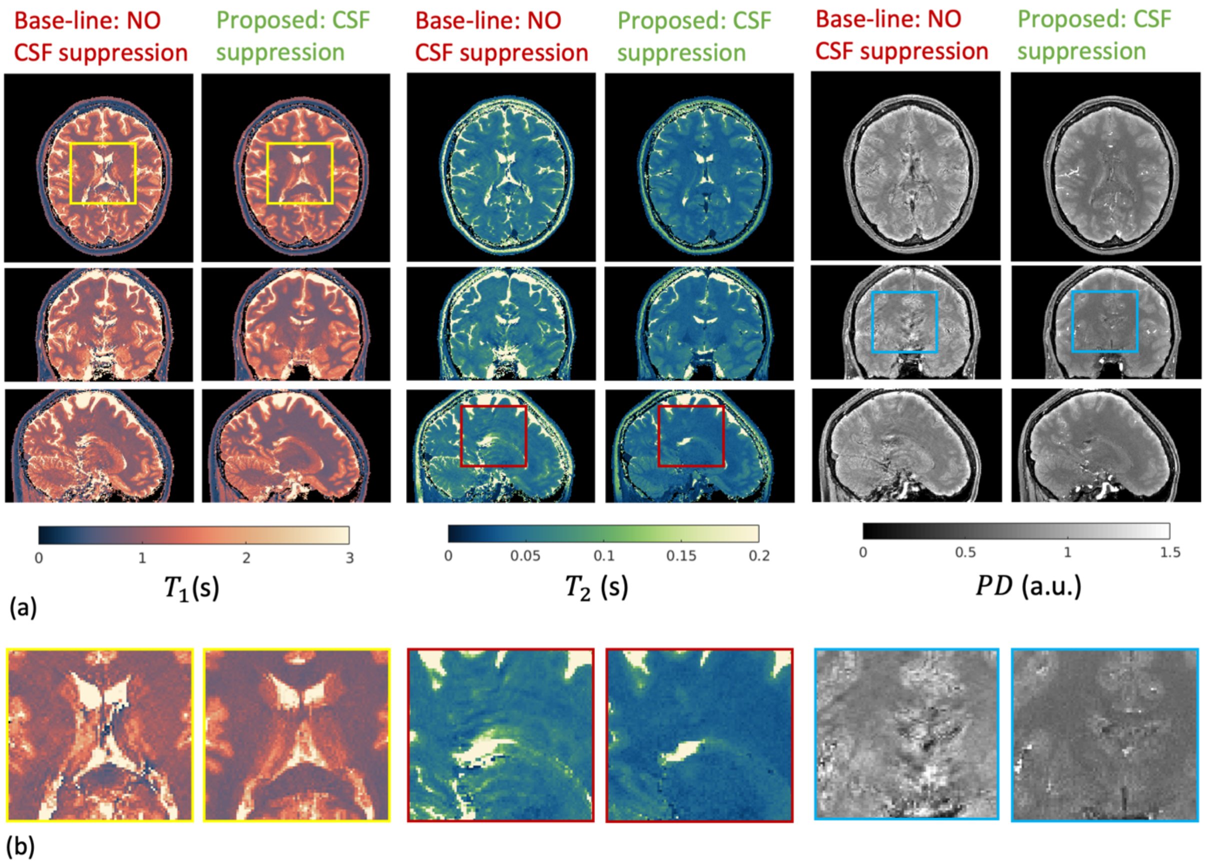

Figure S9. The SENSE reconstruction results after reconstruction sub-step 1 for volunteer no.1. Image-domain results of the central slice for each of the five k-spaces are shown here for both sequences, showing the effectiveness of the CSF suppression for the proposed sequence, similar as **Figure 2(a)**.

For the proposed sequence, a CSF-to-WM ratio larger than one (though still much lower than in the baseline sequence) is observed in only one of the five k-spaces (the first one). In contrast, relatively high CSF-to-WM ratios are observed in three of the five k-spaces (1st, 4th, and 5th) for the baseline sequence.

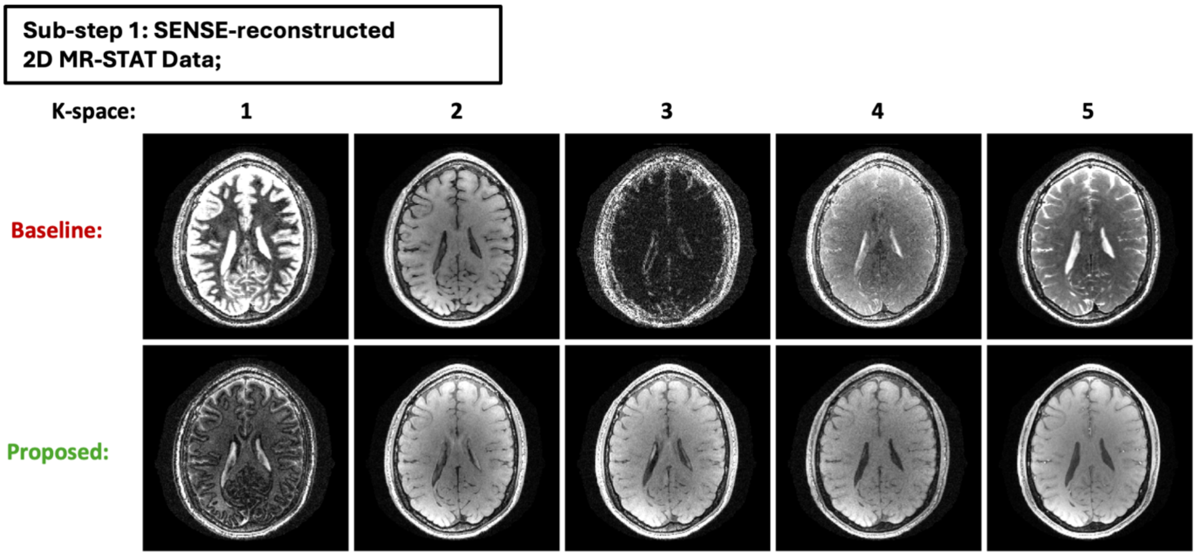

Figure S10. In-vivo 3D MR-STAT results using the baseline and the proposed CSF-suppressed flip-angle trains. Representative transverse and sagittal slices of quantitative maps are shown for volunteer no. 6. Traditional contrast-weighted images acquired from 3D T1w-TSE and 3D T2-FLAIR protocols are also acquired and presented as anatomical references.

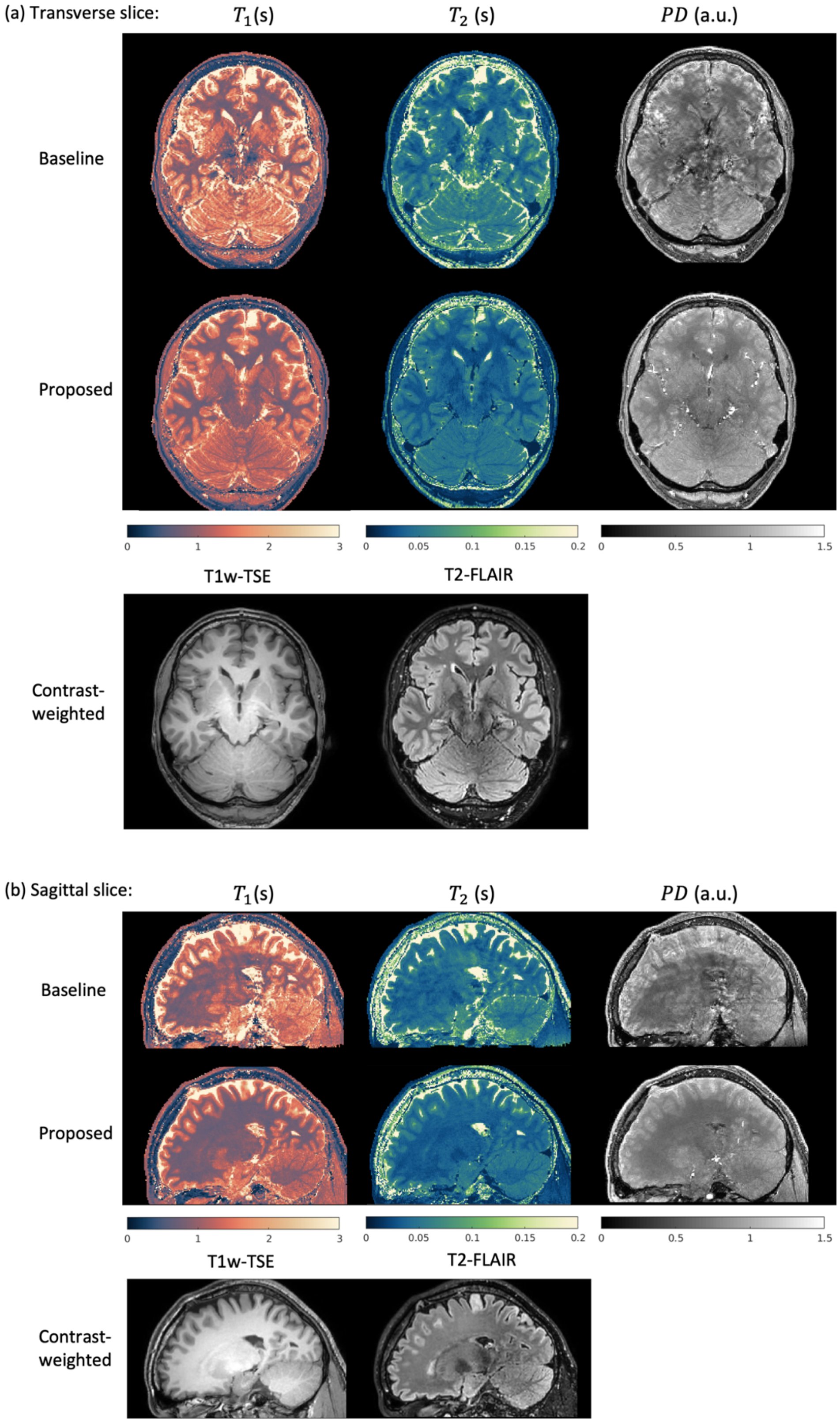

Figure S11. Animation of whole 3D volume quantitative maps for Volunteer no. 1 using the proposed new sequence. (See separately uploaded mp4 file).

Figure S12. Animation of whole 3D volume quantitative maps for Volunteer no. 6 using the proposed new sequence. Conventional contrast-weighted images (3D T1w-TSE and T2-FLAIR) are shown in the second row for reference. (See separately uploaded mp4 file).
